# Again the Great American Fraud

**Published:** 1906-11

**Authors:** 


### AGAIN THE GREAT AMERICAN FRAUD.

COLLIER'S WEEKLY has recently completed the series of articles directed at the exposure of quacks and quackery. The last of the series deals with the varieties of quacks who exploit alleged cures for cancer, eye and ear diseases, drug habits and with some of the minor fry which more unobtrusively, not from modesty but from poverty, attack the sick.

For a long time religious papers, especially those of the type in which God and Mammon are pitted against each other in the literary and the business departments, have carried the ad. of a family named Bye who have in their possession a soothing oil for the cure of cancer. The cancer is gently urged out of existence by the insinuating blandness of the wonderful oil and the despairing patient almost imperceptibly reconducted to a condition of health, cheerfulness and usefulness. Just how many mortals have had their way greased to immortality by this unctuous material is quite beyond conjecture, but the number must be great, for the mortality and frequency of cancer are well established and greatly dreaded and this philanthropic family has been in the business a long time.

It is singularly gratifying to read how these oily scoundrels are revealed in their despicable hypocrisy and spurious humility, in the name of God and humanity stealing the few painfully gathered dollars from the unhappy wretch upon whom a loathsome disease has fastened itself and is hurrying him swiftly to the grave.

Can there be a more tragic picture than that offered by a broken-down preacher, pauperized, yet sending the pitiful handful of money donated by charitable friends to this miserable lump of devil's dung for a bottle of his oil to be fatuously rubbed to the remnant of a nose or the festering slough of what was once a lip. It is enough to wring tears from a Robespierre and if you think it is overdrawn, look about you. But this pious family of Bye has been rudely disturbed from its cloistral security by the questing hand of *Collier's* and held up to public scorn in all its pallid hideousness.

After duly ticketing this pleasant family group for a permanent position in the Rogues' Gallery, the *Weekly* proceeds to harry into the open such blatant fakirs as Oren Oneal (the jolt given him by Collier's probably dislodged the comma after the Irish prefix) Madison and other eye "specialists" who prey upon the unwary and the unfortunate.

In the guise of a patient, information was obtained from these worthies of such a character as fully to establish the fraudulent nature of their claims. The disguise was then thrown off and they were permitted to see that they had been neatly trapped. They may be able to wiggle out, but they will probably not include *Collier's Weekly* in their evening prayers.

The "dope cure" fraternity comes in for a generous measure of attention. The playful little practical joke of giving more morphine for the cure of the morphine habit, which is worked off on those who seek forgetfulness in the drowsy drug, is shown in a tabulated analysis of the most notorious of the advertised cures. We note with pained surprise the presence in this list of one of our cherished home institution.

The effect of these disclosures is being observed in the gradual withdrawal of reputable newspapers and magazines from participation in the great American fraud by denying the quacks advertising space. It is an encouraging sign.

It is said that once a certain Oriental ruler called to him his grand vizier and desired him to phrase a formula that would cover the fate of all human institutions and experiences. The vizier replied: "This, too, shall pass away!" Perhaps when the devil is dead, McCauley's New Zealander from the top of the Washington Monument will see the last of the obscene breed of quacks disappear beneath the waters of oblivion.

